# Scoring ultrasound synovitis in rheumatoid arthritis: a EULAR-OMERACT ultrasound taskforce**—**Part 1: definition and development of a standardised, consensus-based scoring system

**DOI:** 10.1136/rmdopen-2016-000428

**Published:** 2017-07-11

**Authors:** Maria-Antonietta D’Agostino, Lene Terslev, Philippe Aegerter, Marina Backhaus, Peter Balint, George A Bruyn, Emilio Filippucci, Walter Grassi, Annamaria Iagnocco, Sandrine Jousse-Joulin, David Kane, Esperanza Naredo, Wolfgang Schmidt, Marcin Szkudlarek, Philip G Conaghan, Richard J Wakefield

**Affiliations:** 1 Department of Rheumatology, APHP, Hôpital Ambroise Paré, Boulogne-Billancourt, France; 2 INSERM U1173, Laboratoire d’Excellence INFLAMEX, UFR Simone Veil, Versailles-Saint-Quentin University, Montigny le Bretonneaux, France; 3 Centre of Rheumatology and Spine Diseases, Rigshospitalet-Glostrup, Copenhagen, Denmark; 4 Département de Santé Publique, AP-HP, Hôpital Ambroise Paré, Unité de Recherche Clinique, Boulogne-Billancourt, France; 5 INSERM, VIMA U1168, Villejuif, UFR Simone Veil, Versailles-Saint-Quentin University, Montigny le Bretonneaux, France; 6 Rheumatologie und Klinische Immunologie, Park-Klinik Weissensee, Berlin, Germany; 7 National Institute of Rheumatology and Physiotherapy, Budapest, Hungary; 8 Department of Rheumatology, MC Groep Hospitals, Lelystad, Netherlands; 9 Clinica Reumatologica, Università Politecnica delle Marche, Jesi, Italy; 10 Rheumatology Unit, Università di Torino, Torino, Italy; 11 Department of Rheumatology, CHRU de Brest, Brest cedex, France; 12 EA2216, INSERM ESPRI, ERI29, Laboratoire d'Immunologie, Université de Brest, LabEx IGO, Brest cedex, France; 13 Department of Rheumatology, Trinity College, Dublin, Ireland; 14 Rheumatology and Joint Bone Research Unit, Hospital Universitario Fundacion Jimenez Diaz, Madrid, Spain; 15 Medical Centre for Rheumatology, Immanuel Krankenhaus, Berlin, Germany; 16 Department of Rheumatology, University of Copenhagen Hospital, Køge, Denmark; 17 Leeds Institute of Rheumatic and Musculoskeletal Medicine, University of Leeds and NIHR Leeds Musculoskeletal Biomedical Research Unit, Chapel Allerton Hospital, Leeds, UK

**Keywords:** ultrasound, doppler, scoring system, synovitis, EULAR, OMERACT

## Abstract

**Objectives:**

To develop a consensus-based ultrasound (US) definition and quantification system for synovitis in rheumatoid arthritis (RA).

**Methods:**

A multistep, iterative approach was used to: (1) evaluate the baseline agreement on defining and scoring synovitis according to the usual practice of different sonographers, using both grey-scale (GS) (synovial hypertrophy (SH) and effusion) and power Doppler (PD), by reading static images and scanning patients with RA and (2) evaluate the influence of both the definition and acquisition technique on reliability followed by a Delphi exercise to obtain consensus definitions for synovitis, elementary components and scoring system.

**Results:**

Baseline reliability was highly variable but better for static than dynamic images that were directly acquired and immediately scored. Using static images, intrareader and inter-reader reliability for scoring PD were excellent for both binary and semiquantitative (SQ) grading but GS showed greater variability for both scoring systems (κ ranges: −0.05 to 1 and 0.59 to 0.92, respectively). In patient-based exercise, both intraobserver and interobserver reliability were variable and the mean κ coefficients did not reach 0.50 for any of the components. The second step resulted in refinement of the preliminary Outcome Measures in Rheumatology synovitis definition by including the presence of both hypoechoic SH and PD signal and the development of a SQ severity score, depending on both the amount of PD and the volume and appearance of SH.

**Conclusion:**

A multistep consensus-based process has produced a standardised US definition and quantification system for RA synovitis including combined and individual SH and PD components. Further evaluation is required to understand its performance before application in clinical trials.

**Key messages**

**What is already known about this subject?**

- Ultrasound (US) is able to detect synovitis more accurately than clinical examination.
- No consensus existed until now on a single US scoring system for rheumatoid arthritis (RA) clinical trials.

**What does this study add?**

- After exploring the reasons for discrepancies among a large group of experts, this work iteratively developed an international, consensus-based, RA synovitis scoring system evaluating grey-scale and power Doppler components and their combination and demonstrated the system is highly reliable.

**How might this impact on clinical practice?**

- A consensus-based scoring system for scoring synovitis in RA will enable the use of US as an outcome measure instrument in clinical trials.

In recent years, we have witnessed the increasing use of ultrasound (US) as a tool for assessing patients with inflammatory arthritis and in particular, rheumatoid arthritis (RA). In addition to being inexpensive, safe and widely available, US offers the prospect of more accurate assessment of soft tissue inflammation than conventional clinical examination^1 2^ and with the same sensitivity as magnetic resonance imaging (MRI).^3^ In patients with RA, US is helpful in disease monitoring, in aiding prognosis and potentially acting as a treatment end-point.^4–9^ However, despite the increasing interest and its great utility in every day clinical practice, US is still perceived as an operator-dependent technique restricting its use in clinical trials.

Since the Outcome Measures in Rheumatology (OMERACT) US Working Group formulated the first international consensus on US definitions for joint pathologies in RA, a greater degree of homogeneity has been seen in the published literature when defining RA synovitis.^10^ Both grey-scale (GS) and power Doppler (PD) US have been shown to be sensitive to change and predictive of developing arthritis and radiographic structural damage,^4–9^ but no agreement exists on how to grade the detected changes and to what extent both features of the sonographic inflammatory spectrum should be monitored: the morphological changes in GS (effusion and synovial hypertrophy (SH)), the hypervascularity shown by PD or both. The most frequently used approach for scoring synovitis is a semiquantitative (SQ) grading of severity on a scale from 0 to 3, but after the introduction of Doppler US, some scoring systems focused only on the hypervascularity without taking GS changes (especially the SH) into account.^11–13^ Many different definitions for the individual grades have been proposed from individual groups, and there is no widespread consensus on which of these proposed systems should be applied.^14 15^

A standardised definition of synovitis in RA, as well as a consensus-based scoring system, would therefore improve the performance of US as an outcome measure in RA clinical trials. In order to bring standardisation to the definitions of the elementary lesions of synovitis and to the scoring systems, a group of US experts from the OMERACT US Working Group and from a Task Force of European League Against Rheumatism (EULAR), decided to evaluate the existing scoring systems by assessing the baseline agreement among experts and examine how to reduce variability in scoring synovitis in order to develop an improved, consensus-based definition and grading of synovitis. This was done through a series of iterative exercises that comprised both static and patient-based image assessments, the latter permitting assessment of potential variation in image acquisition.

The project was designed as a stepwise process, with agreement at each step obtained before moving forward. The process began in 2005 and was concluded in 2014. We present here the first two steps of this iterative process, which focused on: (1) evaluating the initial agreement of expert sonographers for grading the severity of small joint synovitis using both the preliminary OMERACT definition for synovitis from 2005 (including SH, effusion and PD components)^10^ and the individual sonographers’ ‘usual practice’ scoring system. If widespread disagreement was found, we would proceed to, (2) evaluating the influence of both the applied definition of synovitis and acquisition technique on the reliability of scoring synovitis by developing an algorithm for analysing the discrepancies. A Delphi exercise would subsequently be conducted to obtain new, consensus definition for synovitis, the elementary components defining an US-detected synovitis and develop a novel consensus based scoring system.

## Methods

### Step 1: assessing baseline US reliability

The initial step, performed during a 2-day exercise, aimed at evaluating intraobserver and interobserver reliability for scoring static images and scoring images acquired in real-time while scanning patients.

*Reading static images (day 1).* Static images, representing a broad range of different degrees of synovitis in the metacarpophalangeal (MCP), wrist, proximal interphalangeal (PIP) and metatarsophalangeal joints (MTP) of patients with RA attending the Rheumatology Department of Ambroise Paré Hospital in Boulogne-Billancourt (France) were anonymised by the convenor (MADA). Images were obtained using the preliminary OMERACT definition for synovitis which includes both GS (SH and effusion) and PD findings. Images were acquired according to the EULAR recommendations^16^ with a longitudinal scan obtained using either a dorsal or volar (plantar) view. Seventeen musculoskeletal sonographers (from Denmark, France, Germany, Hungary, Ireland, Italy, Netherlands, Spain, UK and USA) simultaneously but independently scored the images, which were presented randomly presented with 60 s for evaluating each image. No patient information was made available. Participants were asked to score GS and PD using both a binary (presence/absence) and SQ grading from 0 to 3 (normal, minimal, moderate, severe), according to their own daily practice, on a preprinted data collection sheet.

*Acquiring and reading images (day 2).* A practical exercise was then conducted the following day scanning and scoring synovitis. Eight patients with RA^17^ were recruited from the same Rheumatology Department each having only mild to moderate hand deformities in order to eliminate possible acquisition difficulties due to severe structural deformities including ankylosis. The study was conducted in accordance with the Declaration of Helsinki and each participant gave written informed consent. The examinations were performed on the same day, in the same room, using eight identical machines (Technos MPX - Esaote Biomedica, Genoa, Italy) equipped with a 10–14 MHz broadband linear array transducer. The machines were calibrated with identical Doppler settings (frequency of 10.1 MHz, pulse repetition frequency of 750 Hz and Doppler gain of 50–53 dB). In this way, the impact of machines on the results was minimised. Fourteen rheumatologists who participated on the first day, in step 1, participated on the second day; all were blinded to the clinical details of the patients (ie, presence or not of active disease). Each patient was assigned to one machine and the sonographers then rotated from one machine to the next in a predefined sequence with 10 min allocated for scanning and recording the findings on a standard score sheet. In each patient, the second to fifth MCP and second to fifth PIP joints were scanned bilaterally using a GS and PD longitudinal scan in the midline of the joint on both the dorsal and volar aspects. Sixteen MCP joints were scanned twice in order to assess the intraobserver reliability.

### Step 2: analysis of discrepancies for creating consensus definitions and grading of synovitis

Following step 1, any disagreement in static images and in patients related to the definition and scoring of synovitis was analysed. To reduce variability related to the type of joint, the sonographers decided to focus on the MCP joint as the ‘model joint’. In order to clarify sources of disagreement, the sonographers sent to the project convener images of MCP joints, using either palmar or dorsal longitudinal scans, which illustrated different degrees of synovitis severity according to their usual practice. For each image, participants were asked to describe which elementary components (ie, SH and effusion in GS and PD signal) and which level of echogenicity (ie, hypoechoic, hyperechoic or isoechoic signal for both SH and effusion in GS) best described the observed synovitis and which grade they attributed to each of these elements. In this way, it would be clear which US component had the highest impact on the participants’ evaluation of the presence and grading of synovitis.

The sonographers were also asked to describe their usual scanning preferences (eg, dorsal vs volar), as well as whether they reported the presence of PD signal inside or outside the joint. At the same time, they provided details of their usual practice of grading each elementary component (ie, quantitative, binary or SQ (0–3)). Data were analysed in order to retrieve which information produced the highest agreement between participants for describing US-detected synovitis.

The different definitions of synovitis, obtained by the combination of each elementary component as proposed by the experts, as well as the possible grading systems, were then distributed to the participants in a Delphi exercise. Each sonographer had to score, on the proposed definitions, using a 5-level rating scale (1=strongly disagreed to 5=strongly agreed). Only definitions of components and severity grade reaching a consensus of at least 75% were accepted.

## Statistical analysis

The intraobserver and interobserver reliability of scoring static and real-time acquired images were assessed according to kappa (κ) statistics. A weighted κ coefficient was used in order to take into account the magnitude of discrepancy between categories giving different weights to disagreementsaccording to the magnitude of discrepancy. Intraobserver coefficients were evaluated on pairs of measures performed by the same sonographer at each site. Calculation of interobserver coefficient was exclusively based on the first measure of those pairs. Interobserver reliability was studied by calculating the mean κ for all pairs (ie, Light’s κ).^18^ Kappa values of 0–0.20 were considered poor, 0.20–0.40 fair, 0.40–0.60 moderate, 0.60–0.80 good and 0.80–1 excellent according to Landis and Koch.^19^ Statistical analysis was performed using the R software (http://www.r-project.org/).

## Results

### Step 1: baseline agreement

Eighty-six static images were scored and 20 of them were repeated randomly to assess intrareader reliability. Overall, the individual intrareader reliability appeared better with SQ than with binary scoring (tables 1 and 2). As expected for static images, the intrareader reliability for scoring PD activity was found to be excellent for both binary and SQ grading (κ 1 and between 0.89 and 1, respectively), whereas GS reliability showed greater variability (−0.05 to 1 for binary and 0.59 to 0.92 for SQ, respectively).

**Table 1:** Reliability of scoring synovitis in static images and in patients with RA

Intraobserver					Interobserver			
Static images					Static images			
	Prevalence % (min–max)	Observed agreement(min–max)	Kappa (y/n)+95% CI(min–max)	Kappa (0–3)(min–max)	Prevalence % (mean)	Observed agreement(mean)	Mean Kkappa (y/n)+95% CI	Mean Kkappa (0 to 3)+95% CI
GS*	Grade 0: 3.1–17.2	0.66–0.94	−0.05 to 0.94 (0–1 to 0.7–1)	0.59–0.92 (0.1–0.65 to 0.7–0.94)	Grade 0: 11.8	0.77	0.71 (0.16 to 0.73)	0.76 (0.08 to 0.78)
	Grade 1: 4.7–25				Grade 1: 14.0			
	Grade 2: 28.1–57.8				Grade 2: 43.9			
	Grade 3: 6.3–42.2				Grade 3: 30.3			
PD	Grade 0: 57.8–59.4	0.89–1	1–1 (0.89–1 to 1)	0.89–1 (0.7–0.94 to 0.38–1)	Grade 0: 59.3	0.82	0.98 (0.98 to 1)	0.94 (0.92 to 1)
	Grade 1: 1.6–21.9				Grade 1: 7.9			
	Grade 2: 14.1–32.8				Grade 2: 22.4			
	Grade 3: 6.3–18.8				Grade 3: 10.4

**Table:** 

Patients					Patients			
	Prevalence % (min–max)	Observed agreement (min–max)	Kappa (y/n) +95% CI (min–max)	Kappa (0–3) +95% CI (min–max)	Prevalence % (mean)	Observed agreement (mean)	Mean Kappa (y/n) +95% CI	Mean Kappa (0 to 3) +95% CI
GS*	Grade 0: 15.6–68.8	0.53–0.88	0–0.94 (0–0 to 0.56–1)	0.05–0.83 (0.04–0.28 to 0.59–0.89)	Grade 0: 38.5	0.67	0.31 (0.13 to 0.48)	0.43 (0.18 to 0.44)
	Grade 1: 16.7–49.5				Grade 1: 30.2			
	Grade 2: 10–53.2				Grade 2: 23.6			
	Grade 3: 0–33.3				Grade 3: 7.7			
PD	Grade 0: 75–89.6	0.66–0.94	0.2–0.91 (0–0 to 1–1)	0.2–0.89 (0–0.54 to 0–1)	Grade 0: 81.0	0.82	0.42 (0.17 to 0.63)	0.42
	Grade 1: 3.1–18.8				Grade 1: 10.6			
	Grade 2: 2.1–16.7				Grade 2: 7.3			
	Grade 3: 0–5.2				Grade 3: 1.1			

Kappa coefficients for assessing the sonographers reliability to detect and scoring synovitis in patients with RA using the OMERACT definition of synovitis (*GS synovitis (synovial hypertrophy, with or without effusion) and PD signal) in static images (on the top of the table) and during live scanning of patients with RA (at the bottom of the table). For the intrareader reliability, the kappa range is listed.  GS, grey-scale; OMERACT, Outcome Measures in Rheumatology; PD, power Doppler; RA, rheumatoid arthritis.

**Table 2:** Inter-readers reliability of scoring synovitis in static images according to the joints

Joints	GS* Mean kappa		PD Mean kappa	
	(yes/no) +95% CI (min–max)	(0–3) +95% CI (min–max)	(yes/no) +95% CI (min–max)	(0–3) +95% CI (min–max)
MCP	0.74	0.83	0.97	0.92
MTP	0.52	0.66	0.99	0.93
PIP	0.59	0.74	1.0	0.92
Wrist	0.74	0.74	0.97	0.91

Kappa coefficients for assessing the sonographers reliability to detect and scoring synovitis using the OMERACT definition of synovitis (*GS synovitis (synovial hypertrophy, with or without effusion) and PD signal) in patients with rheumatoid arthritis, on static images.

GS, grey-scale; MCP, metacarpophalangeal joint; MTP, metatarsophalangeal joint; OMERACT, Outcome Measures in Rheumatology; PD, power Doppler; PIP, proximal interphalangeal joint.

The inter-reader reliability showed more variable results (table 1). Mean κ coefficients for PD scoring (for both binary and SQ) were again higher than for GS scoring (0.98 and 0.94, respectively for PD and 0.71 and 0.76 for GS).

When the reliability was analysed according to the individual type of joint (only inter-reader), a higher reliability was observed for GS scoring of MCP’s and wrist joints for both binary and SQ grading (0.74–0.83 for binary and SQ scoring of MCP, respectively and 0.74 for both binary and SQ for wrist) (table 2). For PD, the κ coefficients overall were excellent irrespective of the joint site (0.91–1). However, although the mean κ coefficients according to the joint were higher with PD than with GS (0.91–1 and 0.52–0.83, respectively), discrepancies were found between binary and SQ grading. For PD, the κ coefficients were higher with binary than SQ (0.98 and 0.94, respectively), but for GS, it was the opposite with SQ being higher than the binary scoring (0.76 and 0.71 respectively).

The reliability for scanning patients was quite variable. The κ coefficients for the intraobserver reliability were overall low for both modalities and for both types of scoring. In GS, binary scoring varied from 0 to 0.94 and SQ from 0.05 to 0.83. Using PD, the binary scoring varied from 0.2 to 0.91 and 0.2–0.89 for SQ scoring (tables 1 and 3). The mean κ coefficients for overall interobserver reliability never reached 0.5 for both modalities (table 1), again lower for GS than PD.

**Table 3:** Reliability of scoring synovitis in patients according to the joints and scanning position

	GS* (yes/no)	GS* (0–3)	PD (yes/no)	PD (0–3)
Inter-readers reliability (mean)				
MCP	0.39	0.53	0.49	0.50
PIP	0.11	0.18	0.16	0.18
Scanning position				
** Volar**	0.32	0.45	0.30	0.31
Dorsal	0.28	0.40	0.51	0.48

Kappa coefficients for assessing the participants’ reliability to detect and score synovitis using the OMERACT definition of synovitis (*GS synovitis (synovial hypertrophy, with or without effusion) and PD signal) in rheumatoid arthritis, when performing.

GS, grey-scale; MCP, metacarpophalangeal joint; OMERACT, Outcome Measures in Rheumatology; PD, power Doppler; PIP, proximal interphalangeal joint.

When evaluating the reliability according to the type of joint (only interobserver), higher κ values were seen for GS scoring of the MCP joints (0.39–0.53 for binary and SQ, respectively) than for PIP joints (0.11–0.18 for binary and SQ, respectively) (table 3). Similar results were found for PD.

When considering the dorsal vs the volar approach for both MCP and PIP altogether, the mean κ values varied from 0.32 to 0.45 for GS (binary and SQ, respectively) in the volar aspect and from 0.28 to 0.51 (for binary and SQ, respectively) in the dorsal aspect (table 3). Additional data are reported in the online supplementary material.

### Step 2: development of new definitions and grading of elementary components of synovitis

The analysis of disagreement observed in step 1 and the first two rounds of the Delphi exercise showed that some of the differences encountered in scoring synovitis were related to different weightings attributed to each elementary component used for defining synovitis (ie, hypoechoic or hyperechoic SH and detection of PD inside–outside the SH), which also impacted the grading of its severity. Participants’ definition and scoring of each single component in GS (ie, SH and effusion) did not differ significantly by using the volar or dorsal scan of the joint. However, for the detection of PD activity, all (100%) agreed that the dorsal scan appeared more sensitive. Consequently, the dorsal evaluation of the MCP was agreed as the key scanning position for image acquisition of synovitis (90%). A perfect agreement (100%) during the first round was reached on the following definitions:

1. A normal joint is one with no hypoechoic SH, regardless of the presence of effusion, and without PD signal detected within the synovium.
2. GS synovitis is hypoechoic SH regardless of the presence of effusion and any grade of PD signal.
3. A positive PD signal is at least one red spot within the hypoechoic SH.

Greater than 90% participants agreed not to consider effusion alone (ie, without concomitant SH) as a sign of synovitis, and not to define and score joint effusion and SH together as components of a common process (GS synovitis). They agreed to define synovitis as ‘hypoechoic SH’ even in the absence of PD signal (>90%). This decision was made based on the consideration of the huge variability of the Doppler modules across different US machines, some of them having a poor PD sensitivity even in presence of an acceptable GS. They also agreed (86%) to use a 0–3 score for each elementary component (ie, SH and PD signal, the SQ grading of PD being a modified version of the PD SQ scoring system proposed by Szkudlarek) allowing more Doppler to be present in Grade 1.^11^

**Figure 1:**
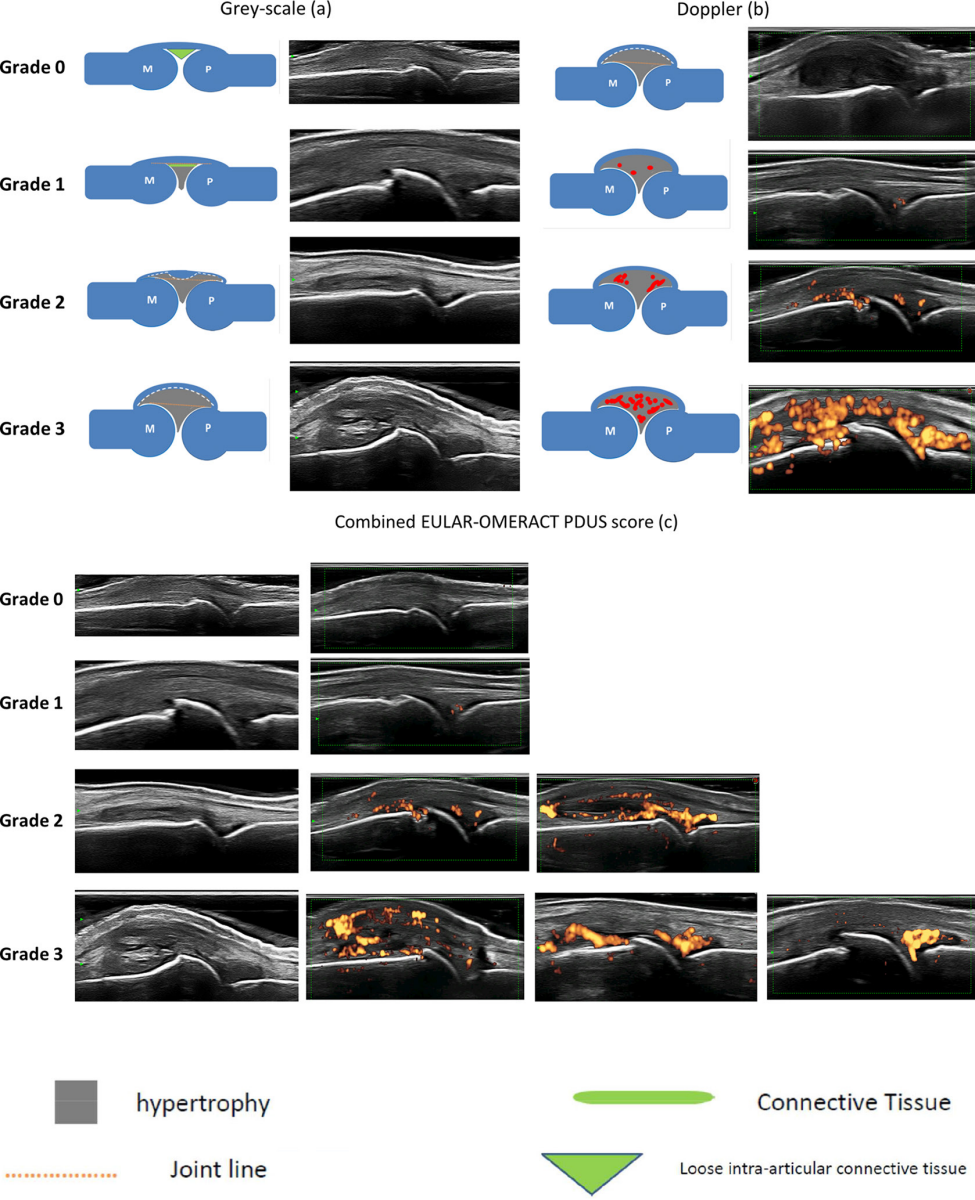
(Panel 3a shows the schematic drawing of the individual grades of hypoechoic SH for GS alone. For each grade is also shown the corresponding GS image. (1) None=Grade 0: no SH independently of the presence of effusion; (2) minimal=Grade 1: SH with or without effusion up to level of horizontal line connecting bone surfaces M and P; (3) moderate=Grade 2: SH with or without effusion extending beyond joint line but with upper surface convex (curved downwards) or hypertrophy extending beyond joint line but with upper surface flat; (4) severe=Grade 3: SH with or without effusion extending beyond joint line but with upper surface flat or convex (curved downwards). Panel 2b shows the schematic drawing of the individual grades for Doppler activity. For each grade is also shown the corresponding ultrasound image. (1) None=Grade 0: no Doppler activity; (2) minimal=Grade 1: up to three single Doppler spots or up to one confluent spot and two single spots or up to two confluent spots; (3) moderate=Grade 2: greater than Grade 1 but <50% Doppler signals in the total GS background; (4) severe=Grade 3: greater than Grade 2 (>50% of the background GS). Panel 2c shows the EULAR-OMERACT score for PDUS synovitis combining grey-scale SH and PD signal. Normal joint=Grade 0: no grey-scale-detected SH and no PD signal (within the synovium); minimal synovitis=Grade 1: Grade 1 SH and ≤Grade 1 PD signal; moderate synovitis=Grade 2: Grade 2 SH and ≤Grade 2 PD signal or Grade 1 SH *and* a Grade 2 PD signal; severe synovitis=Grade 3: Grade 3 SH and ≤Grade 3 PD signal or Grade 1 or 2 synovial hypertrophy *and* a Grade 3 PD signal. fx1, connective tissue; EULAR, European League Against Rheumatism; GS, grey-scale; fx2, hypertrophy; fx3, joint line; fx4, loose intra-articular connective tissue; M, metacarpal head; P, proximal phalangeal bone; OMERACT, Outcome Measures in Rheumatology; PD, power Doppler; SH, synovial hypertrophy.

Based on the points above, more than 90% of the participants agreed on assessing and grading synovitis by using GS SH and PD signal together in a combined SQ scoring system (the ‘combined score’). The overall synovitis severity, in the combined score, depends on the amount of PD and the amount and configuration of SH (ie, SH appearing hypoechoic and creating a convex/concave or linear bulging of the capsule profile). In this combined score, the higher of the hypoechoic SH or PD scores is used for grading the overall synovitis severity (table 4). Figure 1a–c shows a schematic drawing with corresponding US images of the agreed grades of SH (figure 1a) and PD (figure 1b) as well as of the combined PDUS score (figure 1c).

**Table 4:** EULAR-OMERACT combined scoring system for grading synovitis in rheumatoid arthritis

Grade 0: Normal joint	No GS-detected SH and no PD signal (within the synovium)
Grade 1: Minimal synovitis	Grade 1 SH and ≤Grade 1 PD signal
Grade 2: Moderate synovitis	Grade 2 SH and ≤Grade 2 PD signal or Grade 1 SH and a Grade 2 PD signal
Grade 3: Severe synovitis	Grade 3 SH and ≤Grade 3 PD signal or Grade 1 or 2 SH and a Grade 3 PD signal

Proposed combined PDUS (GS and PD) scoring system graded from 0 to 3 describing the criteria for the individual grades in relation to the GS SH and Doppler signal. The higher of the two determines the final combined score. EULAR, European League Against Rheumatism; GS, grey-scale; OMERACT, Outcome Measures in Rheumatology; PD, power Doppler; SH, synovial hypertrophy.

## Discussion

We aimed to improve the reliability of US, as several sources of variability needed to be considered, including the theoretical definition of synovitis and the operational definitions of the relevant pathological components, the grading/severity, the machine used and the experience of the operator. The iterative approach used in the process described in this programme of work revealed the detailed reasons for previous differences in scoring synovitis in patients with RA and resulted in new operational definitions and a novel scoring system, applicable to multicentre setting.

In order to obtain a consensus-based scoring system suitable for clinical trials, it was necessary to assess potential baseline disagreements between experts when grading synovitis such as the interpretation and impact of different components in detecting and grading synovitis and the nature of the scanning technique employed.

Several interesting results were found when assessing the baseline agreement. First, we found a relatively good overall reliability between observers when reading and grading static images when applying the original OMERACT definition for synovitis. The initial qualitative disagreement between experts was related to a difference in which elementary lesions should be included in both the definition and scoring of an US-detected synovitis and was demonstrated by a higher variation in interobserver than intra-observer reliability. This indicates that individually the participants knew how they perceived the definition and scoring of synovitis, but that their definition was not necessarily the same as the other participants. In addition, the reliability diminished considerably when the acquisition of images was included in the scoring of synovitis.

The reproducibility of the scanning technique is also complex, as it is related to a number of additional factors including the interaction among the machine, the patient and the operator. Patient factors may include joint deformity and structural damage. To eliminate the possible impact of joint deformities on reliability, only patients with minimal to moderate joint deformities were invited to participate. Subluxation, though rarely seen now, may complicate the grading of synovitis as the capsule is stretched. The variation in Doppler sensitivity in different machines and the subsequent impact on scoring of PD activity was underlined in the paper by Torp-Pedersen *et al*.^20^ The impact of the machines was diminished by using the same machines, with the same settings and in a standardised scanning environment. Consequently, the baseline variation found was reasonably believed to be related to the acquisition technique and to the interpretations of findings.

We also found that the acquisition and grading of PD activity was more reliable than GS findings alone and finally, that a binary scoring system surprisingly was not more reliable than a SQ scale independent of the type of joint evaluated. This may partly be explained by a more sensitive evaluation of the synovial lining when applying a SQ grading rather than a binary evaluation. For the SQ scoring, it is possible to indicate even minimal changes which may not necessarily be pathological; however, this possibility is to some extent lost when only presence/absence of SH can be indicated.

It was not surprising that the overall reliability for scoring GS was lower than for scoring PD. There are several possible explanations. First, defining ‘normal’ synovium is often difficult as joints without RA may exhibit low levels of GS abnormality^21 22^ and variation exists as to what an individual describes as abnormal or ‘within normal range’. In contrast, Doppler activity rarely occurs in normal joints, in particular the MCP and PIP joints.^23 24^ Second, observing colours on images is easier than assessing GS shades, where distinction between different soft tissues may be challenging. It is also possible that both GS effusion and SH are more susceptible to transducer pressure than previously perceived, as the potential effect of transducer pressure is more commonly considered when working with Doppler. Finally, some aspects of the statistical analysis related to the κ method need to be considered, especially the prevalence of the studied lesions.^25^ In our study, all of these sources of variability could have explained our divergent baseline agreement among experts. However, the most important was the lack of agreement on standardised definitions of elementary components and on severity grading. The challenge was therefore to minimise this variability.

In the second step of the standardisation process and based on the baseline disagreement among experts, the elementary lesions composing synovitis were redefined together with its severity grading for both GS and Doppler—based on the appearance of the pathological process. It was also decided by consensus to eliminate effusion when scoring synovitis. Effusion may be a proxy measure of inflammation, which did not add additional weighting to the definition and severity of an US-detected synovitis. As effusion may be frequent in some joint depending on the weight of the person and level of activity, it was considered important to separate the two components, even if in some situations effusion may indeed be pathological and can be chosen to be scored separately. Recently, there has been a trend to focus predominantly on scoring the hyperaemic part of the inflammatory process, which has been perceived to be the most important and probably the most specific US marker of synovial inflammation.^11–13^ However, by not taking the GS pathology into account, important information is lost. Patients may have a grade 0 in Doppler activity (sometimes due to poor Doppler sensitivity of the machine) but still show severe GS SH in the same joint, and such GS pathology alone is predictive of radiographic progression.^26 27^ Colour Doppler may be used if there is poor PD detection with the available equipment.^20^

The second step allowed the group to define what constitutes ‘synovitis’ taking both GS SH and Doppler signals into account and how their presence may influence the apparent degrees of synovitis. It also allowed both components to be combined in the novel scoring system.

In conclusion, operator-dependent influences of acquiring and reading images provide the greatest error when evaluating synovitis. This is the first study to demonstrate that this variability can be markedly improved through the standardisation of scanning technique as well as standardising the definition and relative importance of GS and Doppler components. Further studies are now required to evaluate the performance of this new scoring system and its applicability to other joints and to assess the added value of SH alone in longstanding disease. 

10.1136/rmdopen-2016-000428.supp1 Supplementary material
