# Supplementary material for: Scoring ultrasound synovitis in rheumatoid arthritis: a EULAR-OMERACT ultrasound taskforce—Part 1: definition and development of a standardised, consensus-based scoring system
Source: RMD Open. 2017 Jul 11;3(1):e000428. doi: 10.1136/rmdopen-2016-000428 (PMC5597799; doi:10.1136/rmdopen-2016-000428)
Supplement: Supplementary material [file rmdopen-2016-000428supp001.docx]

Interobserver-reliability – additional data

Table 1: Inter-observer reliability for the individual joints in static images. 4 = semi-quantitaive score 0-3. 2 = binary score= presence/absence

Table 2. Interobserver reliability (Cohen’s kappa and Light Kappa) in patients for MCP joints 2 – 5.

| MCP | Synovitis 0-3 | Doppler 0-3 | Synovitis binary score | Doppler binary score |
| --- | --- | --- | --- | --- |
| 2 | 0.78 | 0.68 | 0.76 | 0.67 |
| 3 | 0.59 | 0.50 | 0.59 | 0.53 |
| 4 | 0.33 | 0.41 | 0.36 | 0.31 |
| 5 | 0.32 | 0.41 | 0.31 | 0.35 |

Table 3. Interobserver reliability (Cohen’s kappa and Light Kappa) in patients by finger ( MCP and PIPs together)

| Finger | Synovitis 0-3 | Doppler 0-3 | Synovitis binary score | Doppler binary score |
| --- | --- | --- | --- | --- |
| 2 | 0.36 | 0.49 | 0.33 | 0.34 |
| 3 | 0.42 | 0.34 | 0.27 | 0.20 |
| 4 | 0.40 | 0.39 | 0.34 | 0.13 |
| 5 | 0.35 | 0.37 | 0.32 | 0.28 |

Above the result for the MCP and PIPs per finger - similar results were found for MTPs and wrist joints
